# Supplementary material for: Complex formation of APP with GABAB receptors links axonal trafficking to amyloidogenic processing
Source: Nat Commun. 2019 Mar 22;10:1331. doi: 10.1038/s41467-019-09164-3 (PMC6430795; doi:10.1038/s41467-019-09164-3)
Supplement: Supplementary file 1 — Supplementary Information [file 41467_2019_9164_MOESM1_ESM.pdf]

SUPPLEMENTARY INFORMATION

Complex formation of APP with $GABA_B$ receptors links axonal trafficking to amyloidogenic processing

Margarita C. Dinamarca et al.,

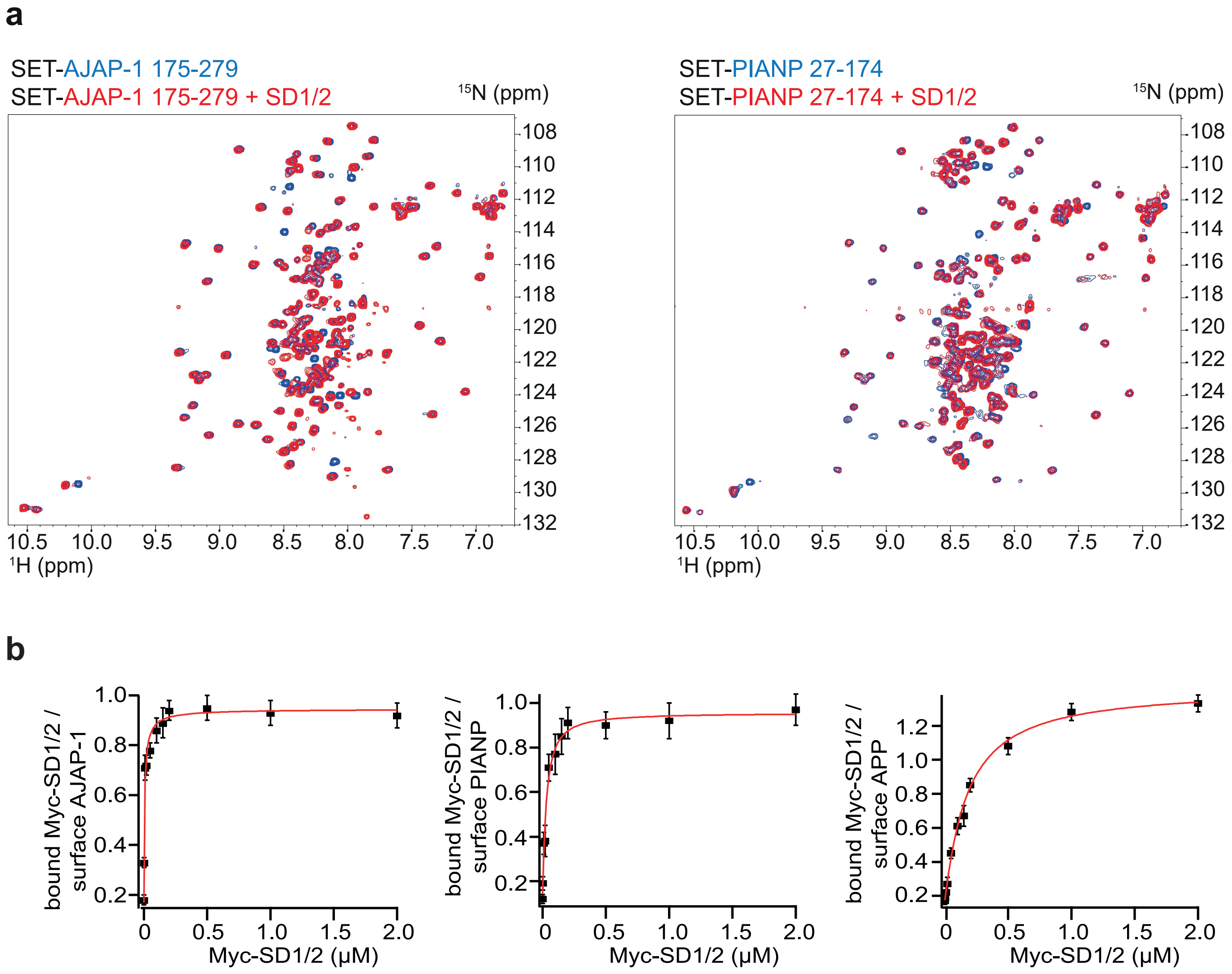

Supplementary Figure 1 AJAP-1, PIANP and APP interact with GB1a via a common WG motif. (**a**) ^{1}H-^{15}N heteronuclear single quantum coherence (HSQC) spectra show complex formation of ^{15}N labeled SET-AJAP-1 175-279 and SET-PIANP 27-174 with unlabeled SD1/2, which identifies a tryptophan-glycine (WG) motif as the central element of the binding interface. The cross peak of the sidechain NH of the respective W disappeared after complex formation with SD1/2. The cross peaks of the solubility enhancement tag (SET = 56-residue B1 domain of streptococcal protein G) were virtually unaffected by the interaction with SD1/2. (**b**) Binding of Myc-SD1/2 to cells expressing APP, AJAP-1 or PIANP was saturable. The estimated apparent dissociation constants (Kd) are 6.4 ± 2.4 nM for AJAP-1, 29.1 ± 5.5 nM for PIANP and 187.6 ± 27.9 nM for APP. Ratio calculated from 3-20 cells for each concentration and fitted by Hill equation with a coefficient of 1. Data are presented as mean ± s.e.m. Source data are provided as a Source Data file.

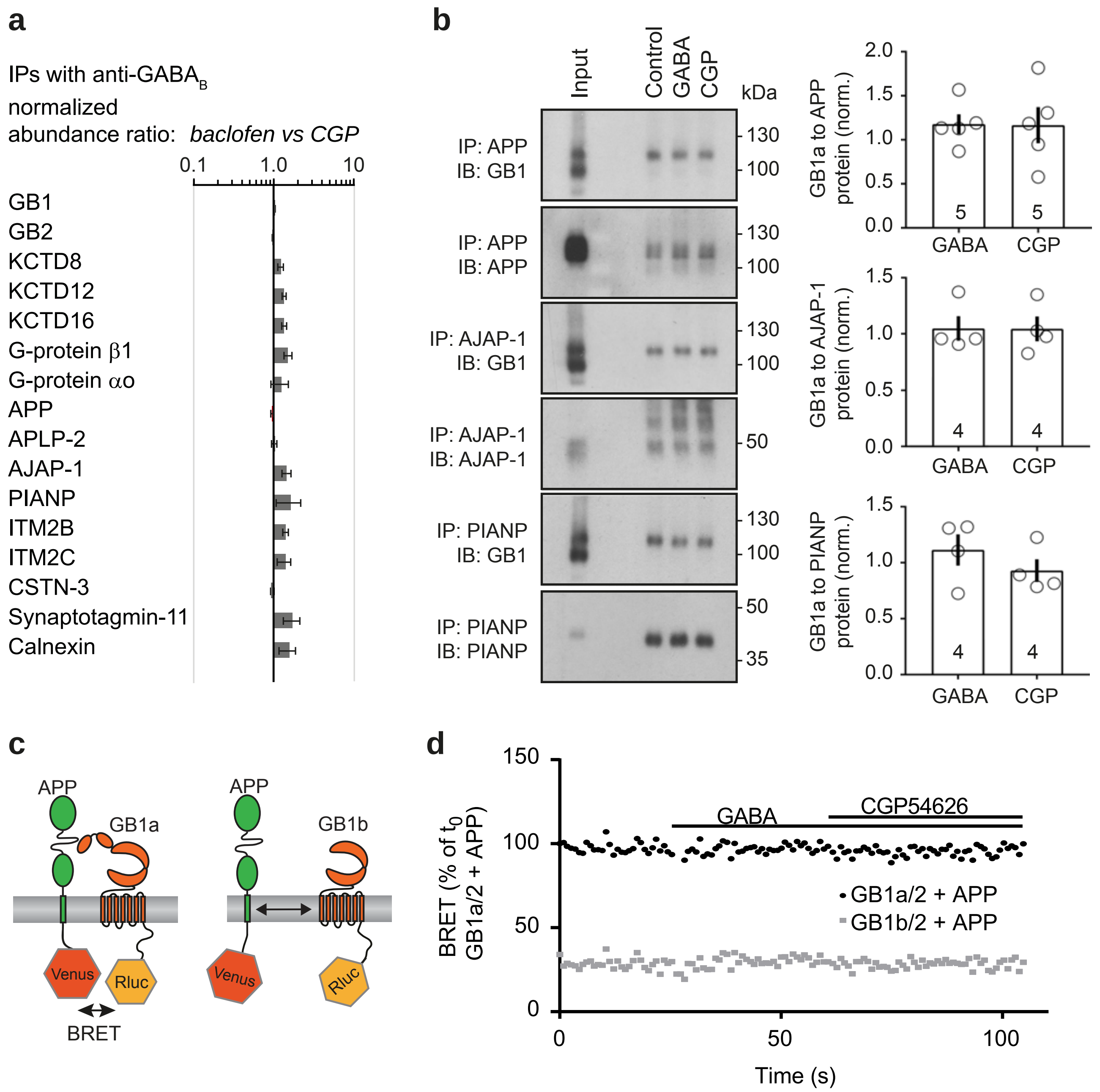

Supplementary Figure 2 GBR activity does not influence the interaction of AJAP-1, PIANP or APP with GB1a. (**a**) Proteomic analysis of native GBR complexes after receptor activation and blockade. Protein abundance ratios for GBR proteome constituents (n = 4 measurements, data are presented as mean ± s.e.m.) in GBR IPs from membrane fractions pre-incubated with baclofen or CGP54626 (normalized to GB1/2). Proteins were solubilized before IP with the intermediate stringency detergent CL91. GBR activity did not influence binding of GB1a-specific interactors (marked in gray). (**b**) Left: Activation or blockade of GBRs with GABA (1 mM) or CGP54626 (CGP, 4 µM), respectively, for 1 hour at room temperature in non-solubilized brain membrane fragments did not significantly alter the amount of GB1a protein co-immunoprecipitating with APP, AJAP-1 or PIANP. IPs from untreated membrane fragments served as controls. Right: Bar graphs summarizing the densitometric quantification of co-immunoprecipitated GB1a protein relative to the immun-precipitated protein. GB1a/APP: control 1.0, GABA 1.18 ± 0.25, CGP 1.17 ± 0.46; GB1a/AJAP-1: control 1.0, GABA 1.05 ± 0.22, CGP 1.04 ± 0.22; GB1a/PIANP: control 1.0, GABA 1.11 ± 0.28, CGP 0.93 ± 0.20 ($P > 0.05$, Tukey's multiple comparison test). Values normalized to control (100%). n = 4-5 mice. Data are presented as mean ± s.e.m. (**c**) Scheme depicting that complex formation between APP-Venus and GB1a-Rluc leads to BRET. GB1b-Rluc serves as a negative control. (**d**) GABA (1 mM) and CGP54626 (25 µM) did not alter BRET between APP-Venus and GB1a-Rluc in HEK293 cells co-transfected with GB2. Representative traces from 3 independent experiments in quadruplicates are shown. Source data are provided as a Source Data file.

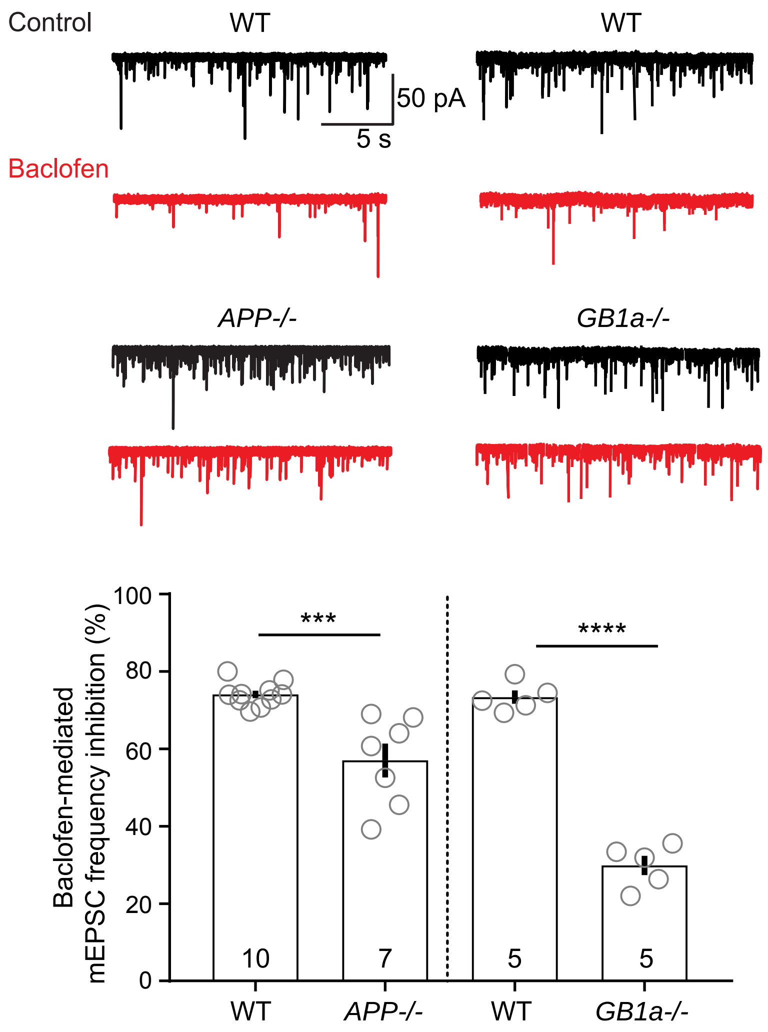

Supplementary Figure 3 Top: Representative mEPSC recordings under baseline conditions (control, black) and during 50 µM baclofen application (red) in cultured days-in-vitro 10 (DIV10) hippocampal neurons of *APP*$^{-/-}$, *GB1a*$^{-/-}$ and WT littermate mice. Bottom: Bar graphs showing that the baclofen-mediated decrease in mEPSC frequency is blunted in *APP*$^{-/-}$ (WT: 74.07 ± 0.98%, *APP*$^{-/-}$: 57.01 ± 4.37%; *** $P < 0.001$, unpaired Student's t-test) and *GB1a*$^{-/-}$ neurons (WT: 73.4 ± 1.7%, *GB1a*$^{-/-}$: 29.8 ± 2.5%; **** $P < 0.0001$, unpaired Student's t-test) compared to WT neurons. Data are presented as mean ± s.e.m. Source data are provided as a Source Data file.

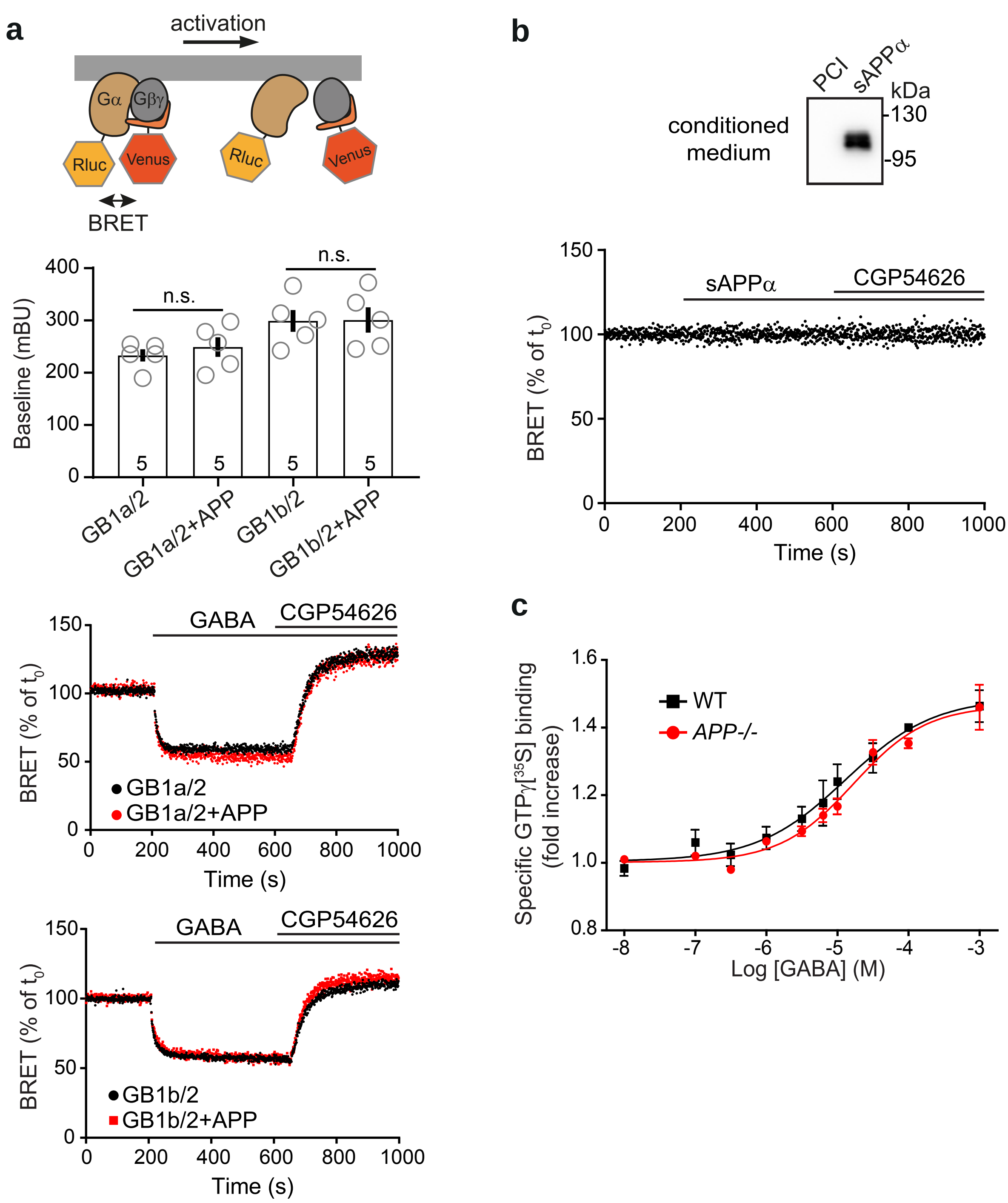

Supplementary Figure 4 APP does not influence receptor-induced G-protein activation. (**a**) Top: Scheme showing that conformational changes during G-protein activation reduce BRET between Gαo-Rluc and Venus-Gγ2. Bottom: Bar graphs indicate that APP does not influence baseline BRET in the presence of GB1a/2 or GB1b/2 receptors ($P > 0.05$, one-way ANOVA). GABA (1 mM) induced changes in BRET in HEK293 cells expressing GB1a/2 or GB1b/2 together with Gαo-Rluc, Venus-Gγ2 and Gβ2, in the presence or absence of APP. After receptor blockade with the antagonist CGP54626 (25 μM) the heterotrimeric G protein re-associates and BRET increases. Receptor activation in the presence and absence of APP induced similar BRET changes. Single experiments carried out in parallel are shown (n = 3 independent experiments). (**b**) Immunoblot analysis of conditioned medium of HEK293 cells expressing sAPPα (APP amino acid residues 1–612). Conditioned medium of HEK293 cells transfected with the PCI vector served as a control. Conditioned medium was collected 48h post-transfection and secreted sAPPα detected using the αAPP antibody 22c11. BRET measurements with transfected HEK293 cells expressing GB1a/2 receptors together with Gαo-Rluc, Venus-Gγ2 and Gβ2 reveal no changes in BRET in response to sAPPα-conditioned medium and the receptor antagonist CGP54626 (25 μM). (**c**) [^{35}S]-GTPγS binding to brain membrane preparations of *APP*$^{-/-}$ (red circles) or control WT littermate mice (black squares) upon stimulation with increasing concentrations of GABA. Data are presented as mean ± s.e.m. Source data are provided as a Source Data file.

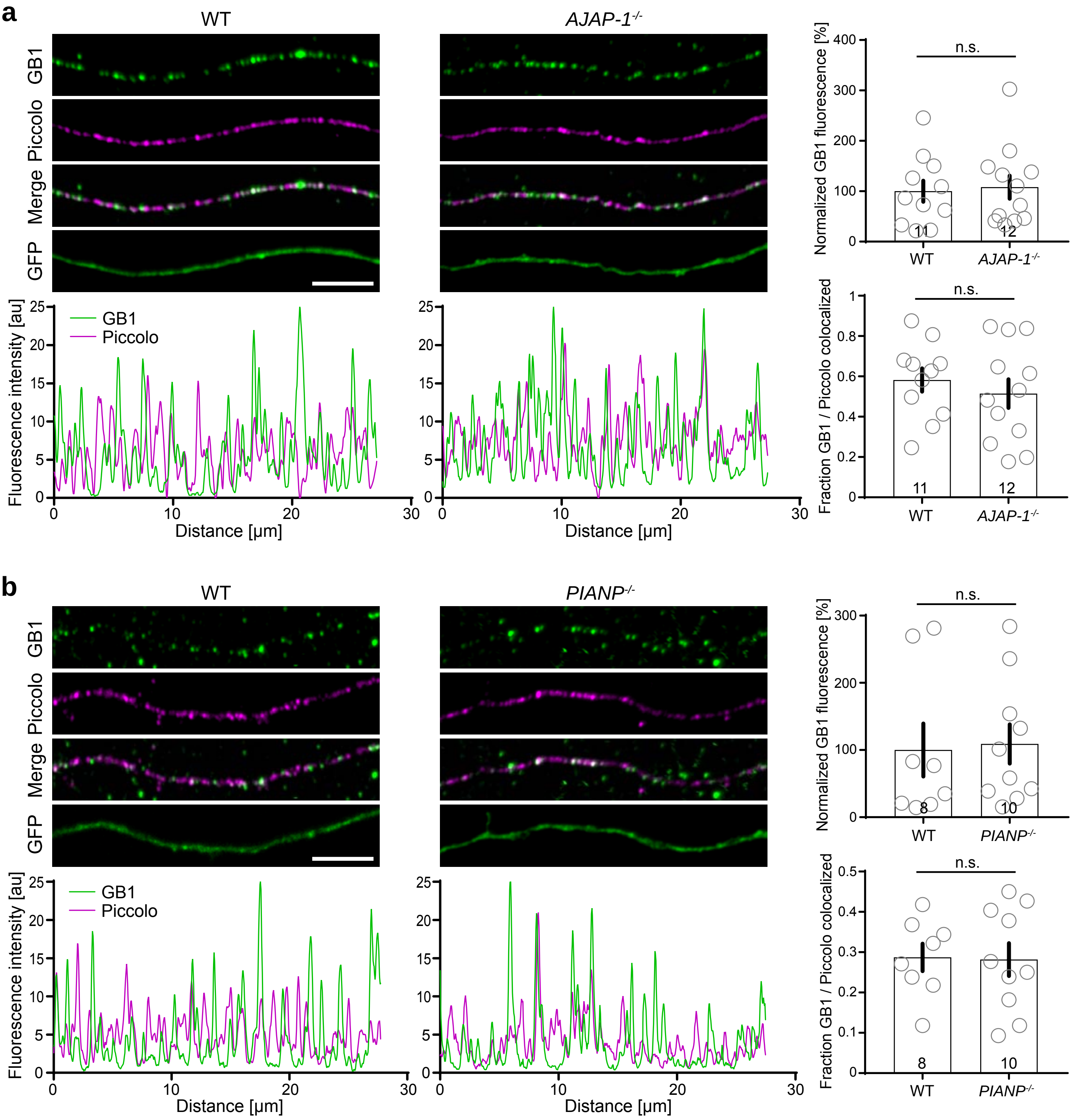

Supplementary Figure 5 Normal axonal GBR expression in *AJAP-1*$^{-/-}$ and *PIANP*$^{-/-}$ mice. (**a**) Top: Immunofluorescence of endogenous GB1 protein in axons of hippocampal *AJAP-1*$^{-/-}$ and WT littermate neurons. Neurons expressing GFP were fixed at DIV10, permeabilized, and immunostained for endogenous GB1 protein (green) and the presynaptic marker piccolo (magenta). GFP served as a volume marker. Merged images show GB1 and piccolo co-localization. Bottom: Intensity grey value profile graphs of GB1 (green) and piccolo (magenta). Normalized GB1 fluorescence refers to the GB1 immunofluorescence intensity normalized to the GFP fluorescence intensity. $P > 0.05$, unpaired t-test. Scale bar 5 µm. (**b**) Immunofluorescence of endogenous GB1 protein in axons of hippocampal *PIANP*$^{-/-}$ and WT littermate neurons. Analysis as in (**a**). Data are presented as mean ± s.e.m. Source data are provided as a Source Data file.

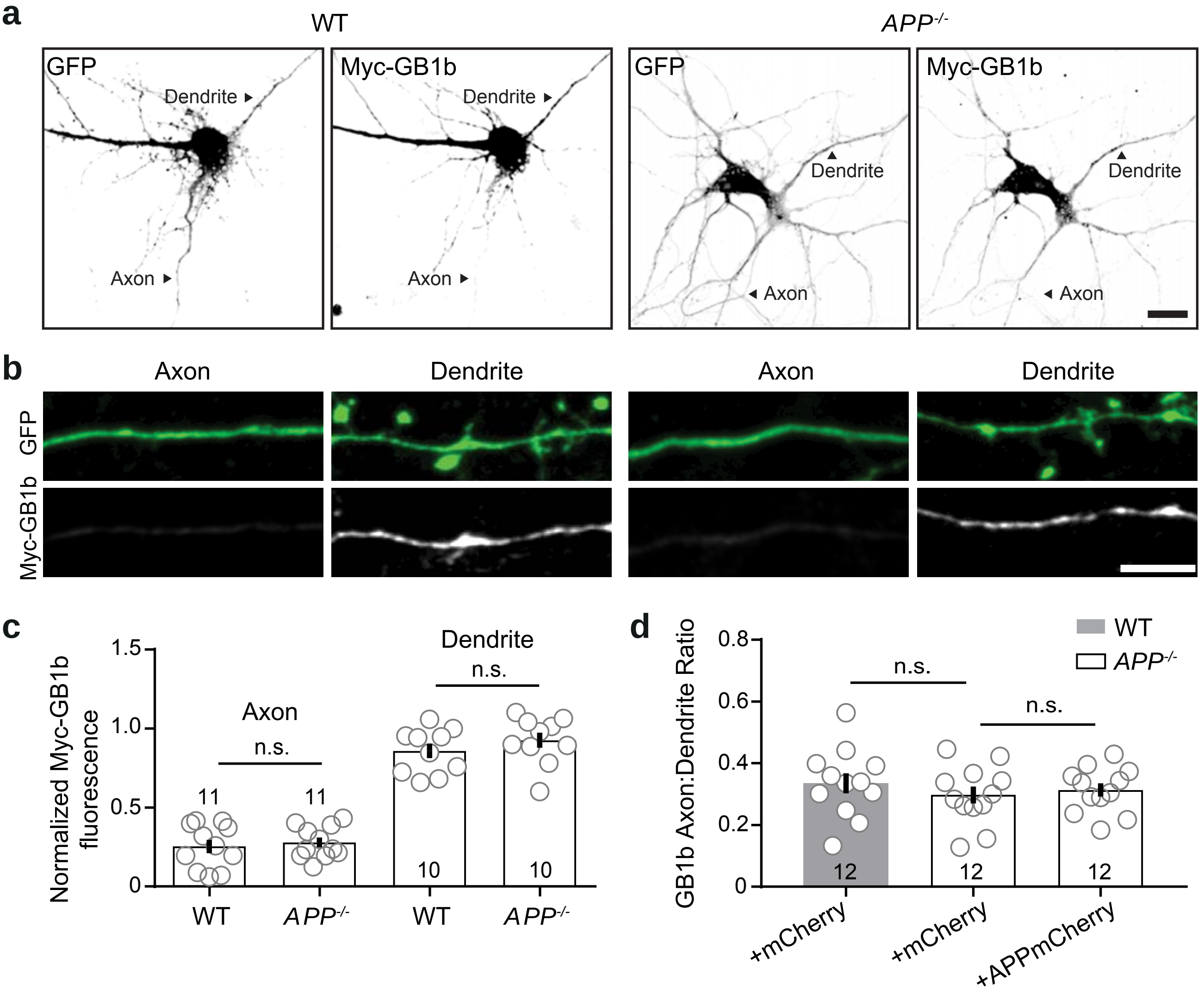

Supplementary Figure 6 APP does not influence Myc-GB1b distribution in cultured hippocampal neurons**.** (**a**) Representative images of neurons co-expressing Myc-GB1b and GFP in *APP*$^{-/-}$ and control WT littermate mice. Transfected neurons were fixed at DIV10, permeabilized, and stained with anti-Myc antibodies. Scale bar 10 µm. (**b**) Higher magnification images of the Myc-GB1b and GFP distribution in distal axons and dendrites of *APP*$^{-/-}$ and WT neurons. Note that Myc-GB1b is present in dendrites but not axons. Scale bar 5 µm. (**c**) Bar graphs showing the normalized Myc-GB1b fluorescence in axons and dendrites of transfected WT or *APP*$^{-/-}$ neurons. Normalized fluorescence refers to the Myc-Gb1b immunofluorescence intensity normalized to the GFP fluorescence intensity (Axon: WT, 0.25 ± 0.04, n = 11, *APP*$^{-/-}$, 0.28 ± 0.03, n = 11, P > 0.05, unpaired t-test; Dendrite: WT, 0.86 ± 0.04, n = 10, *APP*$^{-/-}$, 0.93 ± 0.04, n = 10, P > 0.05, unpaired t-test). (**d**) A:D ratio of Myc-GB1b in *APP*$^{-/-}$ and WT neurons transfected with Myc-GB1b in the presence of mCherry and APPmCherry (DIV10). Note that APPmCherry does not promote axonal localization of Myc-GB1b. P > 0.05, one-way ANOVA. Data are presented as mean ± s.e.m. Source data are provided as a Source Data file.

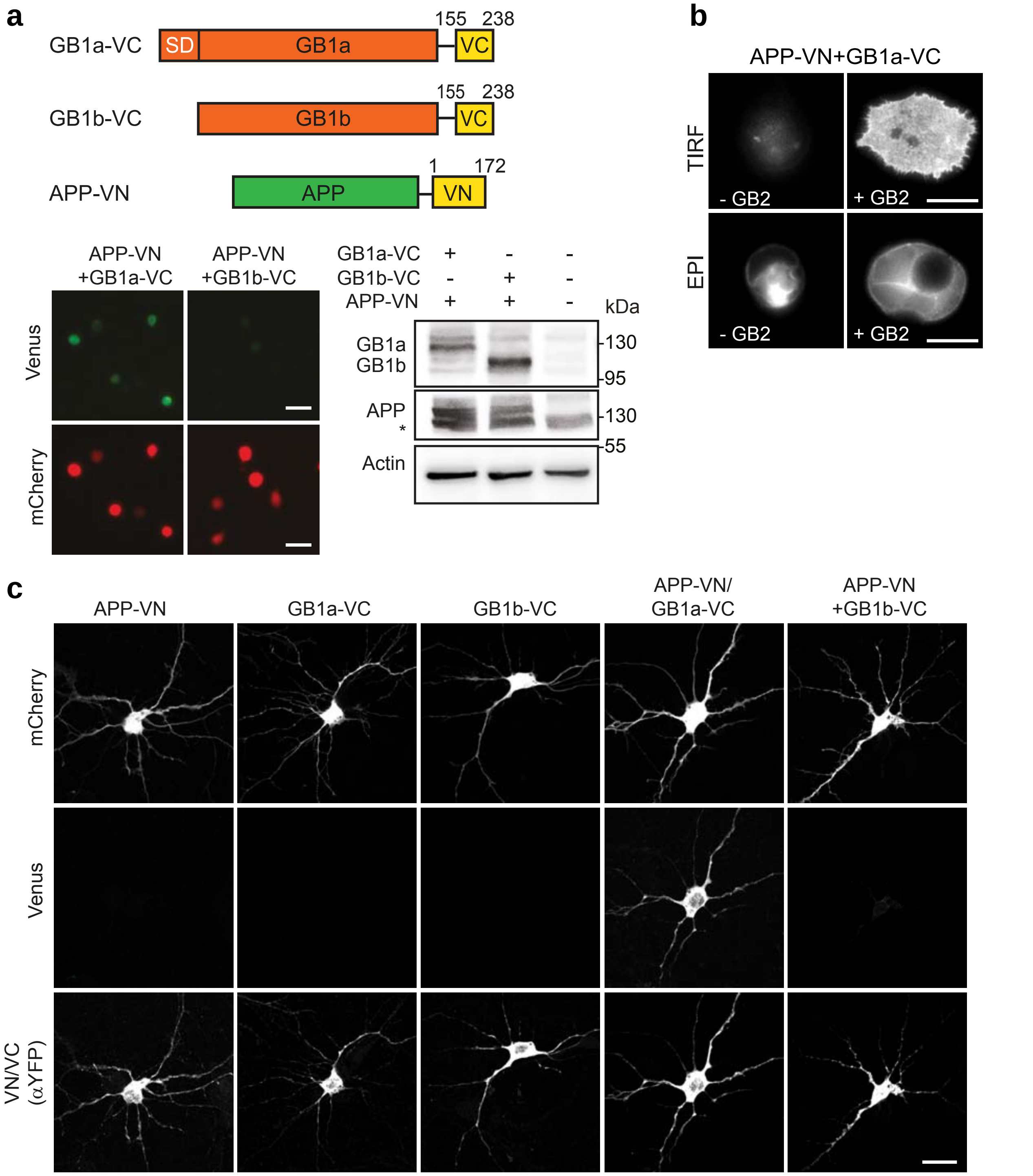

Supplementary Figure 7 The APP-VN/GB1a-VC complex leads to BiFC in transfected HEK293 cells and cultured hippocampal neurons. (**a**) A scheme describing the BiFC constructs is shown on the left. Numbers denote amino-acid residues in Venus. SD, sushi domains; VC, Venus C-terminal domain; VN, Venus N-terminal domain. Images show HEK293 cells expressing GB1a-VC or GB1b-VC together with APP-VN and mCherry as a transfection marker. Cells were analyzed for BiFC of Venus (green) 7-8 hours post-transfection. BiFC with APP-VN is observed for GB1a-VC but not for GB1b-VC. Immunoblotting confirms that cells express similar amounts of GB1a-VC and GB1b-VC. The asterisk indicates endogenous APP. Scale bar 40 µm. (**b**) Representative TIRF and epifluorescence (EPI) images of HEK293 cells expressing GB1a-VC and APP-VN with or without GB2 show that APP/GB1a complexes require GB2 for surface expression. Scale bar 10 µm. (**c**) Expression of GB1a-VC, GB1b-VC and APP-VN alone or in combination in cultured hippocampal neurons. Soluble mCherry served as a volume marker. Neurons were fixed 7-8 hours post-transfection and immunostained with a polyclonal anti-GFP antibody that detects both VC and VN. Note that VC and VN by themselves exhibit no fluores-cence. Scale bar 20 µm. Source data are provided as a Source Data file.

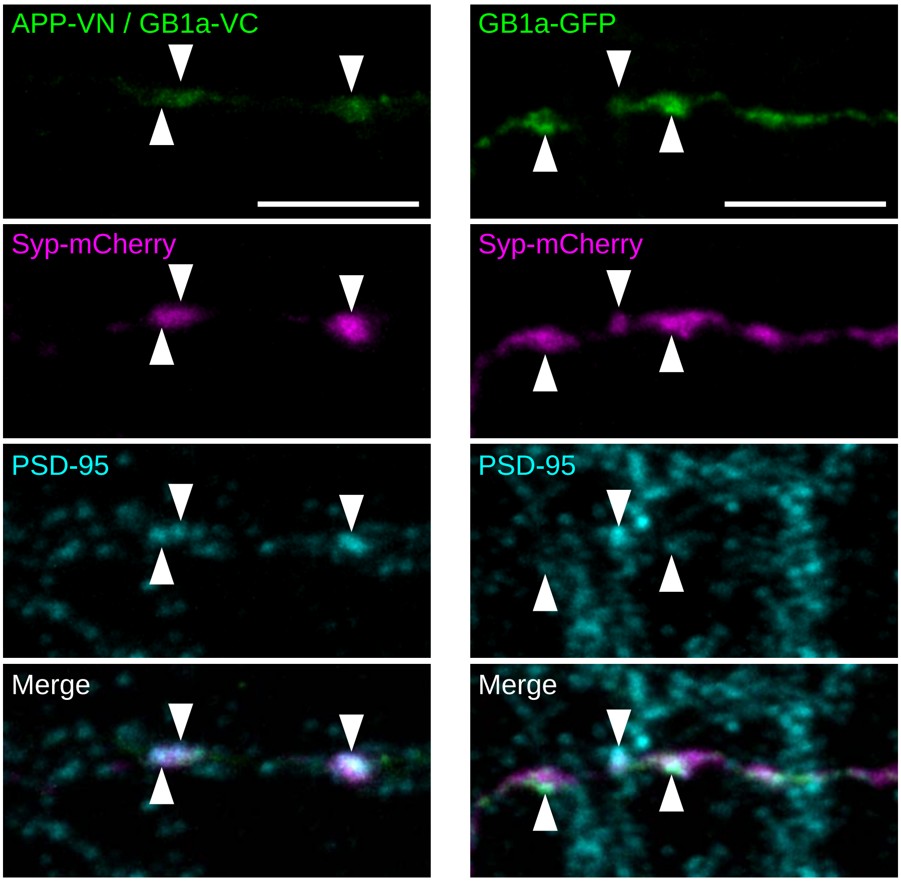

Supplementary Figure 8 The APP-VN/GB1a-VC complex and GB1a-GFP localize to synaptic boutons. Cultured hippocampal neurons were transfected with either APP-VN/GB1a-VC (BiFC) or GB1a-GFP together with Synaptophysin-mCherry. Neurons were fixed at DIV14 and immunolabeled for PSD-95. Synaptic boutons were identified by Synaptophysin-mCherry fluorescence apposed to PSD-95 puncta. Fluorescence for both the APP-VN/GB1a-VC complex and GB1a-GFP were detected at synaptic boutons (arrowheads). Scale bars 5 μm.

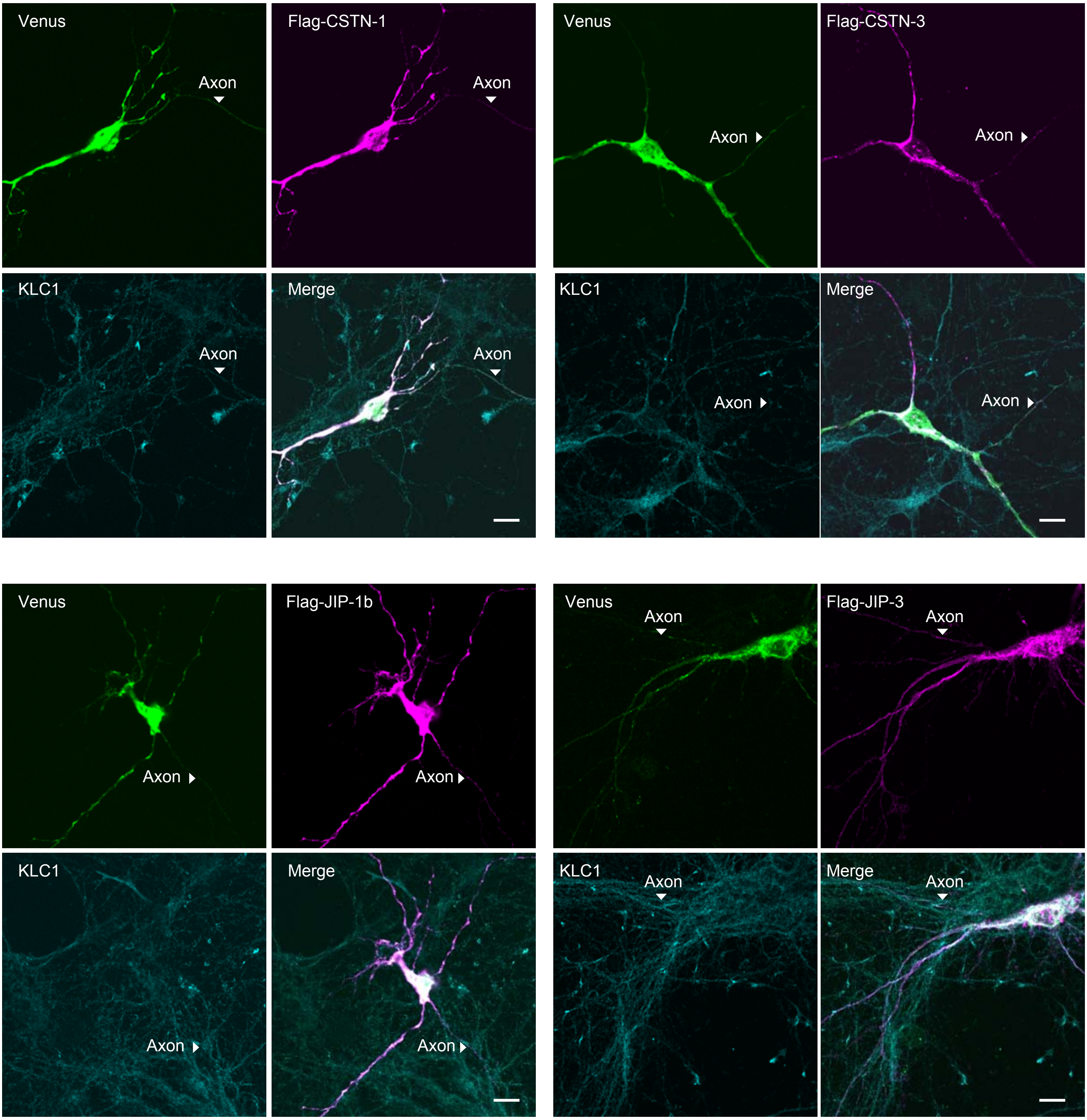

Supplementary Figure 9 The APP-VN/GB1a-VC BiFC complex (green) partly colocalizes with CSTN and JIP in neurons. Co-localization (white, arrowheads) of the APP-VN/GB1a-VC BiFC complex (green) with FLAG-CSTN-1, FLAG-CSTN-3, FLAG-JIP-1b and FLAG-JIP-3 (cyan) and the endogenous kinesin light-chain 1 (KLC1) (blue) in transfected neurons. Note that KLC1 is expressed in axons and dendrites. Scale bar 10 µm. Higher magnification images of axons are shown in **Fig 5c**.

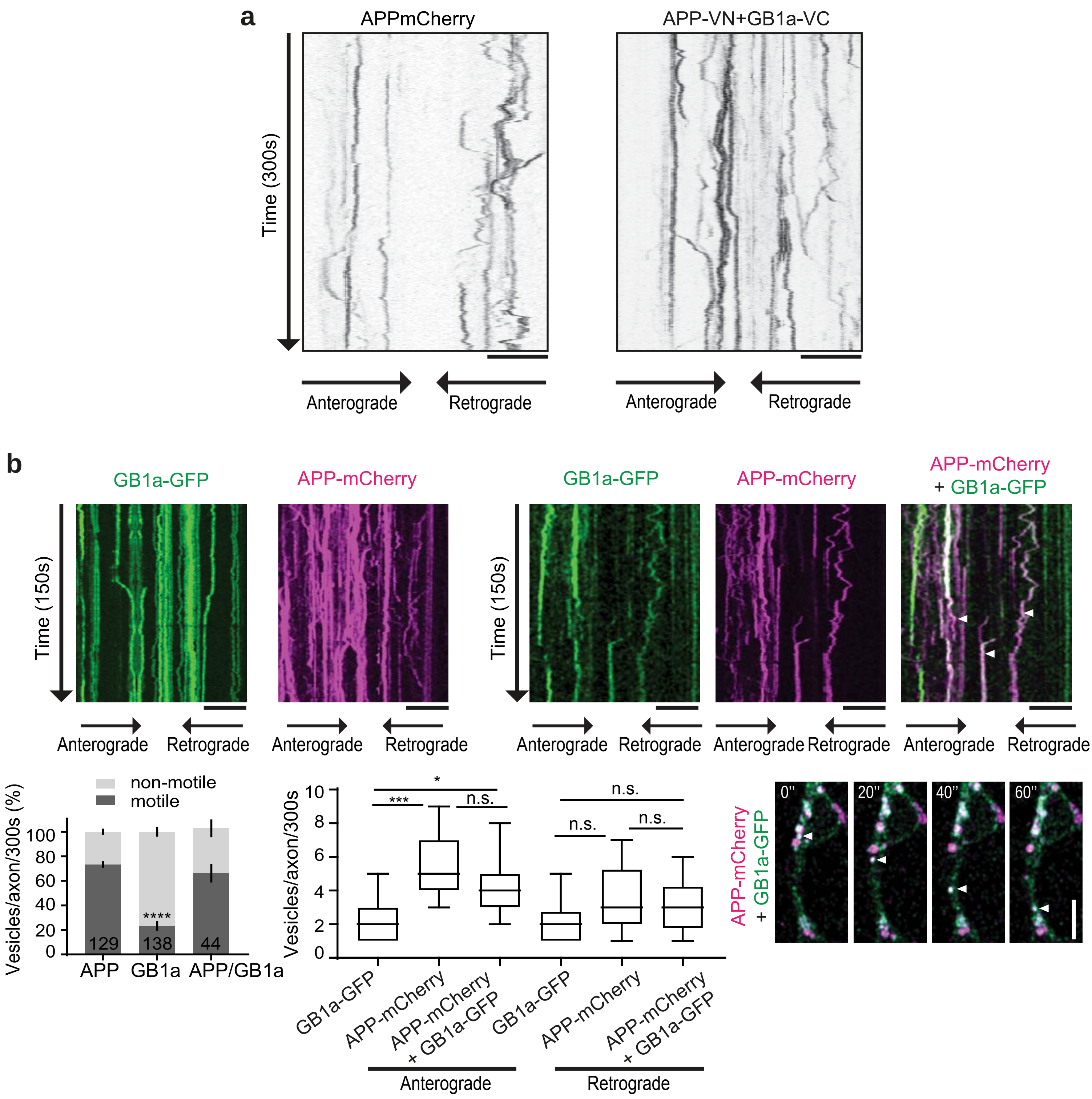

Supplementary Figure 10 Axonal trafficking of APP, GB1a and APP/GB1a complexes in cultured hippocampal neurons. (**a**) Representative kymographs of fluorescent vesicles in axons expressing APPmCherry or APP-VN/GB1a-VC BiFC complexes. Confocal imaging was 12 hours posttransfection at DIV 7 (1 frame/sec). (**b**) Kymographs of vesicles in axons of cultured hippocampal neurons expressing GB1a-GFP, APPmCherry or both. Overlay of kymographs from axons coexpressing GB1a-GFP and APPmCherry identifies APPmCherry/GB1a-GFP complexes (arrowheads). Likewise, time-lapse imaging of axons co-expressing GB1a-GFP and APPmCherry identifies mobile APPmCherry/GB1a-GFP vesicles (arrowheads, acquisition times in seconds). TIRF imaging was 24 hours post-transfection at DIV8. Bar graphs show that fewer GB1a-GFP than APPmCherry or APPmCherry/GB1a-GFP vesicles are mobile. Data are presented as mean ± s.e.m. The number of vesicles moving antero- and retrogradely per axons within 5 min are shown in a min to max - box and whisker plot, with whiskers representing the smallest and largest values, the boxes representing the 25% - 75% percentile and the middle line representing the median. Statistical analysis one-way ANOVA. Scale bars 25 μm. Source data are provided as a Source Data file.

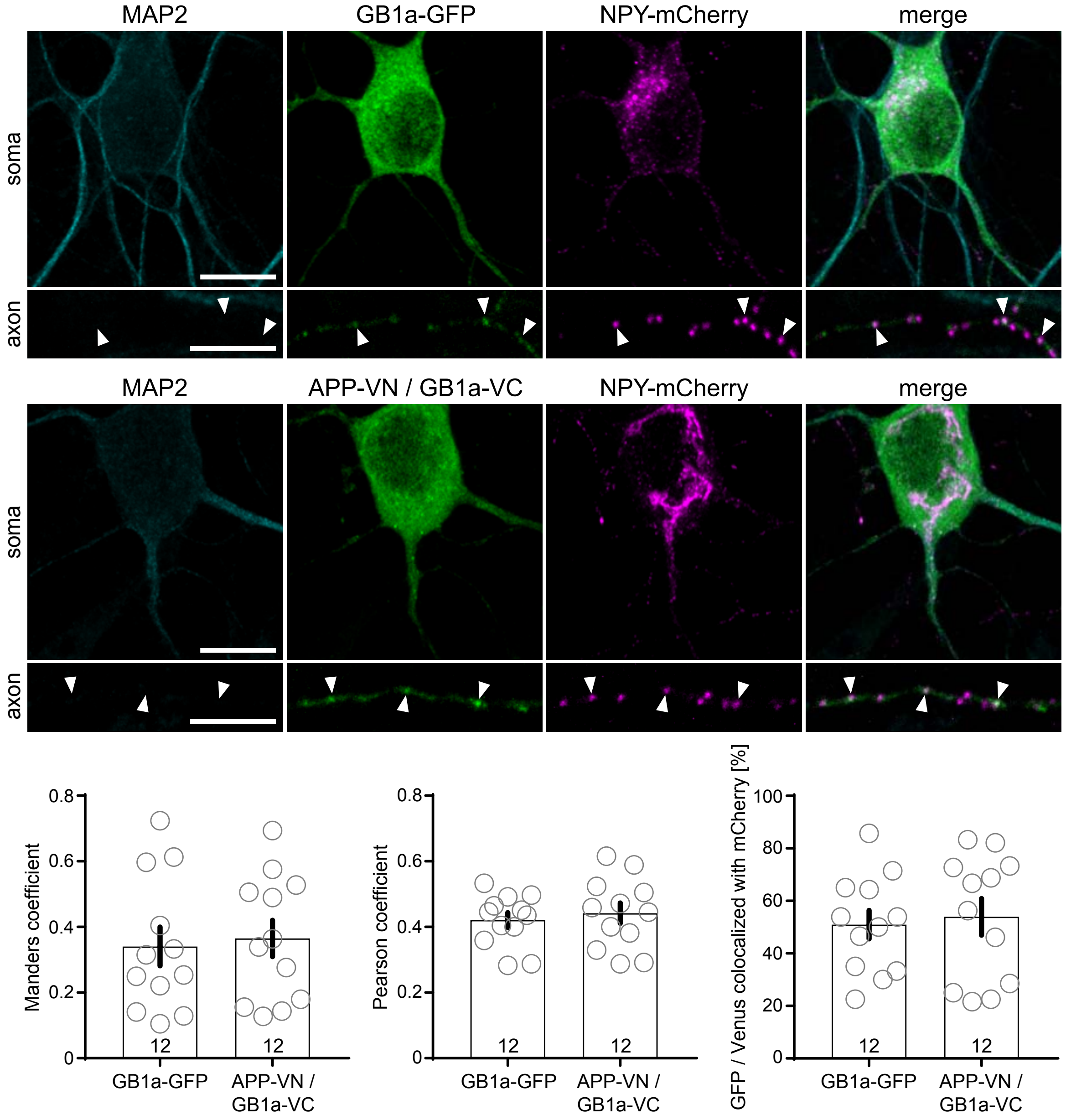

Supplementary Figure 11 NPY-mCherry positive vesicles convey GB1a-GFP and APP-VN/GB1a-VC protein in axons. Hippocampal neurons were transfected at DIV5 and imaged at DIV6. Arrowheads indicate co-localization of NPY-mCherry with GB1a-GFP or APP-VN/GB1a-VC (BiFC). Scale bars 10 µm (top), 5 µm (bottom). Bar graphs indicate the percentage of GB1a-GFP and APP-VN/GB1a-VC positive vesicles that contain NPY-mCherry, as well as the Mander's and Pearson coefficients for co-localization with NPY-mCherry. Data are presented as mean ± s.e.m. Source data are provided as a Source Data file.

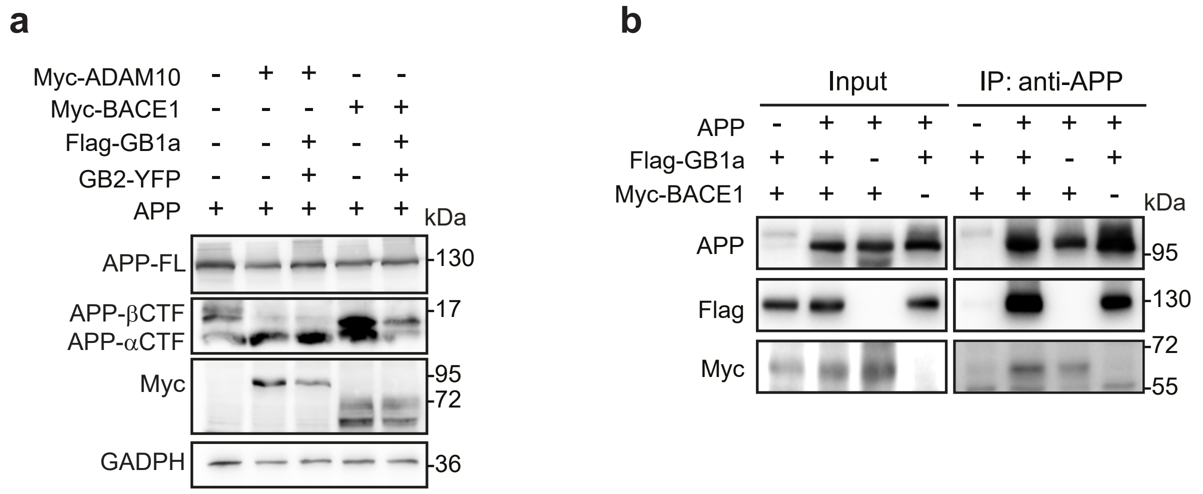

Supplementary Figure 12 GB1a/2 receptors neither prevent interaction of APP with BACE1 nor influence ADAM10 activity. (**a**) Immunoblots of HEK293 expressing APP with or without GB1a/2 receptors together with Myc-BACE1 or Myc-ADAM10. GB1a/2 reduces BACE1 but not ADAM10 proteolysis. APP full length, APP-FL; β-carboxy-terminal fragment of APP, APP-βCTF; α-carboxy-terminal fragment of APP, APP-αCTF. Glyceraldehyde 3-phosphate dehydrogenase (GADPH) served as a loading control. (**b**) Immunoblot of the co-IP of Myc-BACE1 with APP in the presence or absence of Flag-GB1a from transfected HEK293 cells. Note that GB1a does not prevent binding of BACE1 to APP. Source Data are provided as a Source Data file.

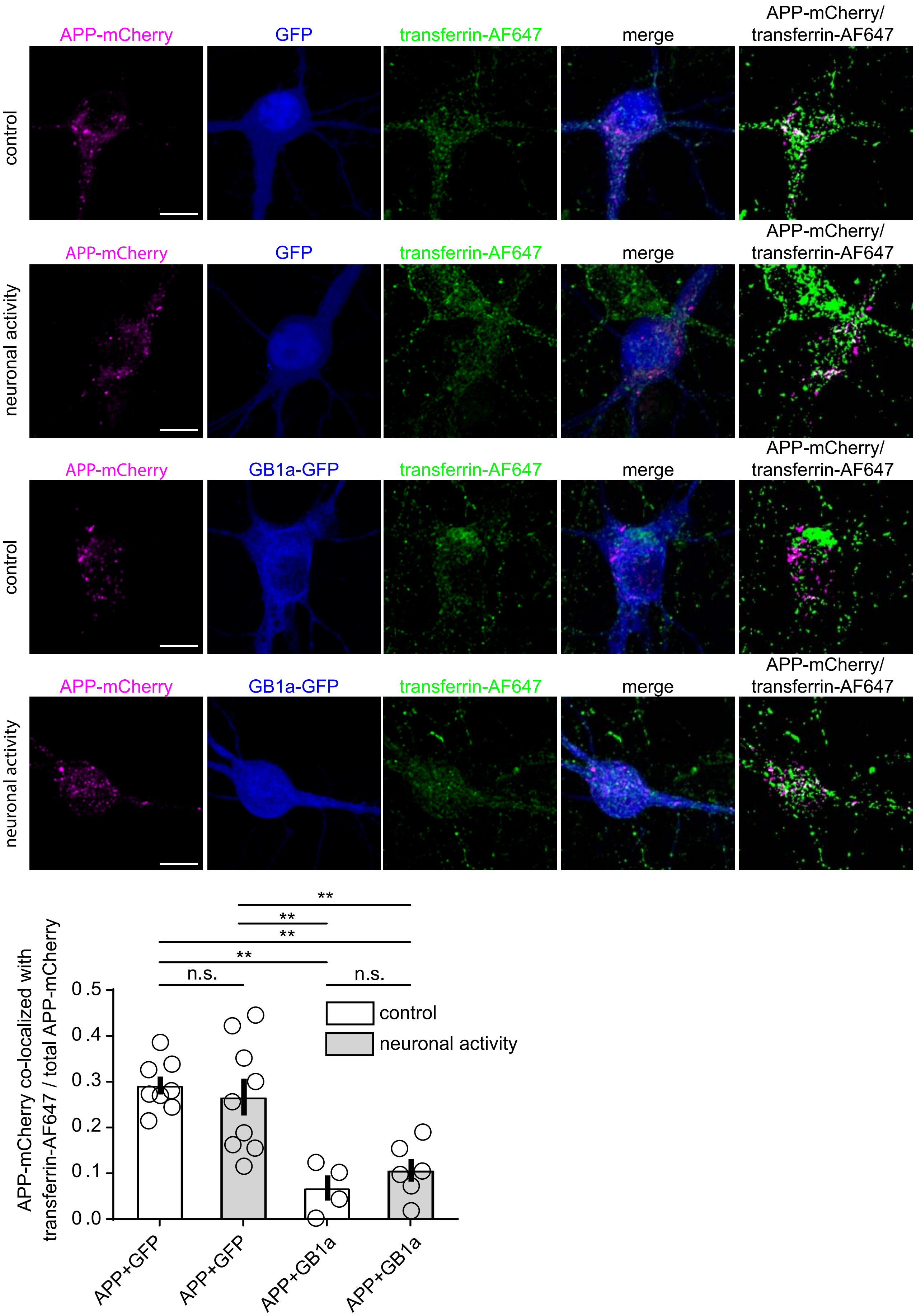

Supplementary Figure 13 Expression of GB1a-GFP in cultured hippocampal neurons decreases localization of APP-mCherry in early endosomes. Transfected hippocampal neurons were incubated with transferrin-AF647 at DIV14 to determine co-localization of APP-mCherry with transferrin-AF647 positive endosomes. Neuronal activity was elicited in parallel cultures using 20 μM bicuculline and 200 μM glycine. GB1a-GFP significantly decreased the presence of APP-mCherry in transferrin-AF647 positive endosomes. Neuronal activity did not significantly alter endosomal localization of APP-mCherry, both in the presence and absence of GB1a-GFP. Binary images indicate the fraction of APP-mCherry (magenta) present in early endosomes (green) as white regions. Scale bars 10 μm. Bar graphs indicate the ratio of APP-mCherry to total APP-mCherry in early endosomes. Statistical analysis was performed with one-way ANOVA and Tukey's multiple comparison test. $^{**}P < 0.01$, $n = 4 - 9$ neurons. Data are presented as mean ± s.e.m. Source data are provided as a Source Data file.
