# Supplementary material for: Complex formation of APP with GABAB receptors links axonal trafficking to amyloidogenic processing
Source: Nat Commun. 2019 Mar 22;10:1331. doi: 10.1038/s41467-019-09164-3 (PMC6430795; doi:10.1038/s41467-019-09164-3)
Supplement: Supplementary file 3 — Description of Additional Supplementary Files [file 41467_2019_9164_MOESM3_ESM.pdf]

Description of Additional Supplementary Files

File Name: Supplementary Movie 1
Description: Live confocal imaging of anterogradly trafficking APP/GB1a complexes in the axon of cultured hippocampal neurons. APP/GB1a complexes were visualized by bimolecular fluorescence complementation of APP-VN with GB1a-VC, which reconstitutes Venus fluorescence.
