# Supplementary material for: Complex formation of APP with GABAB receptors links axonal trafficking to amyloidogenic processing
Source: Nat Commun. 2019 Mar 22;10:1331. doi: 10.1038/s41467-019-09164-3 (PMC6430795; doi:10.1038/s41467-019-09164-3)
Supplement: Supplementary file 5 — Reporting Summary [file 41467_2019_9164_MOESM5_ESM.pdf]

nature research

Corresponding author(s): Bernhard Bettler, Jochen Schwenk

Reporting Summary

Nature Research wishes to improve the reproducibility of the work that we publish. This form provides structure for consistency and transparency in reporting. For further information on Nature Research policies, see Authors & Referees and the Editorial Policy Checklist.

Statistical parameters

When statistical analyses are reported, confirm that the following items are present in the relevant location (e.g. figure legend, table legend, main text, or Methods section).

n/a	Confirmed	
☐	☒	The exact sample size (*n*) for each experimental group/condition, given as a discrete number and unit of measurement
☐	☒	An indication of whether measurements were taken from distinct samples or whether the same sample was measured repeatedly
☐	☒	The statistical test(s) used AND whether they are one- or two-sided<br>*Only common tests should be described solely by name; describe more complex techniques in the Methods section.*
☒	☐	A description of all covariates tested
☐	☒	A description of any assumptions or corrections, such as tests of normality and adjustment for multiple comparisons
☐	☒	A full description of the statistics including central tendency (e.g. means) or other basic estimates (e.g. regression coefficient) AND variation (e.g. standard deviation) or associated estimates of uncertainty (e.g. confidence intervals)
☐	☒	For null hypothesis testing, the test statistic (e.g. *F*, *t*, *r*) with confidence intervals, effect sizes, degrees of freedom and *P* value noted<br>*Give P values as exact values whenever suitable.*
☒	☐	For Bayesian analysis, information on the choice of priors and Markov chain Monte Carlo settings
☒	☐	For hierarchical and complex designs, identification of the appropriate level for tests and full reporting of outcomes
☒	☐	Estimates of effect sizes (e.g. Cohen's *d*, Pearson's *r*), indicating how they were calculated
☐	☒	Clearly defined error bars<br>*State explicitly what error bars represent (e.g. SD, SE, CI)*

Our web collection on statistics for biologists may be useful.

Software and code

Policy information about availability of computer code

Data collection	Fusion FX Chemiluminescence System (Vilber Lourmat, Witec AG), Multiclamp700B amplifier (Molecular Devices), Zen Black/Blue (Zeiss), Xcellence software (Olympus).
Data analysis	ImageJ software (NIH), Digidata 1550B digitizer (Molecular Devices), Fiji analysis software, JACoP and KimographBuilder plugin of ImageJ, GraphPad Prism version 7.0 (GraphPad Software, La Jolla, CA). Adobe Photoshop

For manuscripts utilizing custom algorithms or software that are central to the research but not yet described in published literature, software must be made available to editors/reviewers upon request. We strongly encourage code deposition in a community repository (e.g. GitHub). See the Nature Research guidelines for submitting code & software for further information.

Data

Policy information about availability of data

All manuscripts must include a data availability statement. This statement should provide the following information, where applicable:

- Accession codes, unique identifiers, or web links for publicly available datasets
- A list of figures that have associated raw data
- A description of any restrictions on data availability

Data supporting the findings of this study are available within the paper and in the supplementary information files. The mass spectrometry proteomics data have been deposited to the ProteomeXchange Consortium via the PRIDE partner repository with the dataset identifier PXD012487.

Field-specific reporting

Please select the best fit for your research. If you are not sure, read the appropriate sections before making your selection.

☒ Life sciences ☐ Behavioural & social sciences ☐ Ecological, evolutionary & environmental sciences

For a reference copy of the document with all sections, see nature.com/authors/policies/ReportingSummary-flat.pdf

Life sciences study design

All studies must disclose on these points even when the disclosure is negative.

Sample size	No statistical method was used to predetermine sample sizes, but sample sizes are similar to previous publications (5,6,9).
Data exclusions	No samples or animals were excluded from the analyses.
Replication	Data replicates of each experiment are detailed in the Results (all sections, Figs. 1-7) and Supplementary information.
Randomization	Whenever possible, samples were randomly assigned to experimental groups and processing order.
Blinding	There was no blinding.

Reporting for specific materials, systems and methods

Materials & experimental systems

n/a	Involved in the study	
☒	☐	Unique biological materials
☐	☒	Antibodies
☐	☒	Eukaryotic cell lines
☒	☐	Palaeontology
☐	☒	Animals and other organisms
☒	☐	Human research participants

Methods

n/a	Involved in the study	
☒	☐	ChIP-seq
☒	☐	Flow cytometry
☒	☐	MRI-based neuroimaging

Antibodies

Antibodies used

Affinity purifications from brain membranes:
anti-APP, Ab#1, rabbit anti-APP (A8717, Sigma), Ab#2, rabbit anti-APP (ABIN1741750, Antikörper-online), Ab#3, goat anti-APP (sc-7498, Santa Cruz);
anti-AJAP1, Ab#1, sheep anti-AJAP-1 (AF7970, R&D Systems), Ab#2, rabbit anti-AJAP-1 (HPA012157, Sigma), Ab#3, goat anti-AJAP-1 (sc-163371, Santa Cruz);
anti-PIANP, Ab#1, rabbit anti-PIANP (PAB21925, Abnova), Ab#2, rabbit anti-PIANP (HPA010631, Sigma)
anti-GB antibodies: rabbit anti-GB1 (322102, Synaptic Systems), rabbit anti-GB2 (322203, Synaptic Systems), guinea pig anti-GB2 (322204/5, Synaptic Systems).
IP and immunoblot experiments:
The antibodies used for IP were: rabbit anti-c-myc (C3956, Sigma), mouse anti-c-myc 9E10 (sc-40, Santa Cruz), mouse anti-flag M2 (F1804, Sigma), rabbit anti-flag (F7425, Sigma), rabbit anti-GB1 (rat aa 857-960), rabbit anti-GB2 (322203, Synaptic Systems), rabbit anti-APP Y188 (ab32136, Abcam), sheep anti-AJAP-1 (AF7970, R&D Systems), and rabbit anti-PIANP (NBP1-90541, Novus Biologicals)

The primary antibodies used for immunoblot analysis were: mouse anti-GB1 (ab55051, Abcam), rabbit anti-GB1 Ab26 (rat aa 857-960), mouse anti-GB2 (75-124, NeuroMab), mouse anti-c-myc 9E10 (sc-40, Santa Cruz), rabbit anti-c-myc (C3956, Sigma), rabbit anti-flag (F7425, Sigma), mouse anti-APP A4 22C11 (mab348, Millipore), rabbit anti-APP Y188 (ab32136, Abcam), mouse anti-sAPPα clone 6E10 (SIG-39320, BioLegend), mouse anti-sAPPα clone 2B3 (11088, IBL), rabbit anti-sAPPβ Poly8134 (813401, BioLegend), sheep anti-AJAP-1 (AF7970, R&D Systems), rabbit anti-PIANP (PAB21925, Abnova), rabbit anti-Calnexin (ab75801, Abcam), rabbit anti-β-Actin 13E5 (#4970, Cell Signaling), mouse anti-β-Actin (MAB 1501, Millipore) and mouse anti-GADPH (sc-32233, Santa Cruz).
The secondary antibodies were: HRP-conjugated anti-rabbit (NA9340V, GE Healthcare, UK), anti-mouse (NA9310V, GE Healthcare, UK), anti-sheep (ab7111, Abcam), anti-mouse (sc-2005, Santa Cruz), anti-rabbit (sc-2004, Santa Cruz).
Cell surface binding:
mouse anti-c-Myc 9E10 (11667149001, Roche), goat anti-APP (sc-7498, Santa Cruz), sheep anti-AJAP-1 (AF7970, R&D Systems) or rat anti-HA (HA-PIANP) (clone 3F10, 11867423001, Roche).
Secondary antibodies: (Alexa Fluor® 488 donkey anti-goat IgG, Alexa Fluor® 488 donkey anti-rat IgG, Alexa Fluor® 555 donkey anti-mouse IgG all from Life Technologies).
Immunofluorescence:
Primary antibodies used: mouse anti-GB1 (ab55051, Abcam), rabbit anti-GB1 (ab75239, Abcam), chicken anti-map2 (ab5392, Abcam), mouse anti-c-myc 9E10 (11667149001, Roche), mouse anti-piccolo (142111, Synaptic System), rabbit anti-KLC1 (ab187179, Abcam), rabbit anti-GFP (ab290, Abcam), mouse anti-flag M2 (F1804, Sigma), mouse anti-PSD95 (ab2723, Abcam), mouse anti-PSD95 (124011, Synaptic Systems).
Secondary antibodies used: DyLight™ 405 AffiniPure donkey anti-chicken IgY (Jackson Immunoresearch), Alexa Fluor® 488 donkey anti-rabbit IgG (Life Technologies), Alexa Fluor® 555 donkey anti-mouse IgG (Life Technologies), Alexa Fluor® 555 donkey anti-rabbit IgG (Life Technologies), Alexa Fluor® 568 donkey anti-rabbit IgG (Invitrogen), Alexa Fluor® 647 donkey anti-mouse IgG (Invitrogen), Alexa Fluor® 647 donkey anti-chicken IgY (Millipore).

Validation	Validation data are detailed in the Results (all sections, Figs. 1-7) and supplementary information.

Eukaryotic cell lines

Policy information about cell lines

Cell line source(s)	HEK293 from ATCC (CRL-1573)
Authentication	HEK293 cell line was authenticated by ATCC
Mycoplasma contamination	The cell line was not re-tested for mycoplasma contamination since purchase from ATCC.
Commonly misidentified lines (See ICLAC register)	We did not use any commonly misidentified cell lines.

Animals and other organisms

Policy information about studies involving animals; ARRIVE guidelines recommended for reporting animal research

Laboratory animals	Mouse lines used in this study are indicated in Methods section "Mouse strains".
Wild animals	No wild animals were used.
Field-collected samples	No field-collected samples were used.
